# Manipulation and Optical Detection of Artificial Topological Phenomena in 2D Van der Waals Fe_5_GeTe_2_/MnPS_3_ Heterostructures

**DOI:** 10.1002/advs.202207617

**Published:** 2023-06-16

**Authors:** Xiaodie Chen, Haoyun Wang, Manshi Li, Qinghua Hao, Menghao Cai, Hongwei Dai, Hongjing Chen, Yuntong Xing, Jie Liu, Xia Wang, Tianyou Zhai, Xing Zhou, Jun‐Bo Han

**Affiliations:** ^1^ Wuhan National High Magnetic Field Center and Department of Physics Huazhong University of Science and Technology Wuhan 430074 P. R. China; ^2^ State Key Laboratory of Materials Processing and Die & Mould Technology School of Materials Science and Engineering Huazhong University of Science and Technology Wuhan 430074 P. R. China; ^3^ School of Elementary Education Wuhan City Polytechnic College Wuhan 430074 P. R. China

**Keywords:** 2D magnetic heterostructure, artificial topological phenomena, current, magnetic domains, reflective magnetic circular dichroism, van der Waals materials

## Abstract

2D ferromagnet is a good platform to investigate topological effects and spintronic devices owing to its rich spin structures and excellent external‐field tunability. The appearance of the topological Hall Effect (THE) is often regarded as an important sign of the generation of chiral spin textures, like magnetic vortexes or skyrmions. Here, interface engineering and an in‐plane current are used to modulate the magnetic properties of the nearly room‐temperature 2D ferromagnet Fe_5_GeTe_2_. An artificial topology phenomenon is observed in the Fe_5_GeTe_2_/MnPS_3_ heterostructure by using both anomalous Hall Effect and reflective magnetic circular dichroism (RMCD) measurements. Through tuning the applied current and the RMCD laser wavelength, the amplitude of the humps and dips observed in the hysteresis loops can be modulated accordingly. Magnetic field‐dependent hysteresis loops demonstrate that the observed artificial topological phenomena are induced by the generation and annihilation of the magnetic domains. This work provides an optical method for investigating the topological‐like effects in magnetic structures and proposes an effective way to modulate the magnetic properties of magnetic materials, which is important for developing magnetic and spintronic devices in van der Waals magnetic materials.

## Graphical abstract

2D ferromagnet is a good platform to investigate topological effects and spintronic devices. Herein, interface engineering and an in‐plane current are used to modulate the magnetic properties of the nearly room‐temperature 2D ferromagnet Fe_5_GeTe_2_. An artificial topology phenomenon is observed in the Fe_5_GeTe_2_/MnPS_3_ heterostructure which is induced by the generation and annihilation of the magnetic domains.

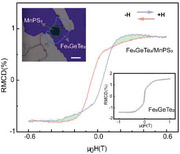

## 1 Introduction

Since the discovery of 2D van der Waals (vdW) magnets, significant interest in 2D magnets has emerged, inspired by their appealing physical properties and integration ability with other 2D materials to form artificial heterostructures. 2D vdW heterostructures provide an alternative bond‐free integration strategy without lattice and processing constraints, forming sharp atomic interfaces with vdW interaction between different layers. These advantages minimize chemical modification and interfacial damage, which is desirable for engineering a clean interface for optimal interactions.^[^ ^1^, ^2^, ^3^, ^4^, ^5^, ^6^, ^7^ ^]^ Among all interface‐engineered heterostructures based on vdW layered systems, the breaking of the inversion symmetry at the interface gives rise to a strong interfacial Dzyaloshinskii–Moriya interaction (DMI), which may change the magnetic domain states and form topologically protected magnetic structures, such as skyrmions or spin spirals.^[^ ^8^, ^9^, ^10^, ^11^, ^12^, ^13^, ^14^, ^15^ ^]^ These have been reported in magnetic multilayers,^[^ ^16^ ^]^ topological insulators,^[^ ^14^ ^]^ and heavy metal/ferromagnet systems.^[^ ^17^ ^]^ Furthermore, with a variety of vdW magnetic materials, the magnetic domain structure can be efficiently tuned by a current or the nearest neighbor materials, thereby yielding some new physical phenomena.^[^ ^18^ ^]^

Topological Hall Effect (THE) is a spin‐chirality‐driven hall effect and the humps or dips near the magnetic phase transition in the hall signal were considered as a feature of the nontrivial spin texture. However, without direct imagination of the chiral magnetic structures by using magnetic force Microscopy or Lorentzian transmission electron microscopy, it is hard to identify the magnetic skyrmions by hallmark alone. The coexistence of the two components’ anomalous Hall Effect may also give rise to the nonmonotonic hysteresis shape that is similar to genuine THE, making it difficult to recognize the genuine THE.^[^ ^19^, ^20^ ^]^ Reflective magnetic circular dichroism (RMCD) is an optic technique that is usually used to characterize the magnetism of 2D magnets. The non‐contact and non‐destructive measurement features and the wavelength‐ and spatial‐resolution abilities made it to be a powerful method for investigating the magnetism of 2D magnets without disturbing their intrinsic properties.

Recent works have demonstrated that Fe_5_GeTe_2_ is a potential candidate for the development of novel room‐temperature spintronic devices,^[^ ^21^, ^22^ ^]^ whose Curie temperature (*T*_c_) in the 2D limit is close to room temperature.^[^ ^23^, ^24^ ^]^ However, due to the complex atomic structure, the magnetic behaviors of Fe_5_GeTe_2_ have not yet been clearly explored. Therefore, a systematic study of the magnetic properties of Fe_5_GeTe_2_ is needed. In this work, we systematically investigated the magnetic properties of Fe_5_GeTe_2_ and Fe_5_GeTe_2_/MnPS_3_ heterostructures. Interface engineering and in‐plane current application could tune the magnetic properties of Fe_5_GeTe_2_ in opposite ways. When a proper in‐plane current was applied to the Fe_5_GeTe_2_/MnPS_3_ heterostructures, an anomalous RMCD hysteresis loop with a hump‐like feature similar to that of THE hysteresis loops could be observed. However, the minor‐loop measurements confirm that this anomalous RMCD signal is not a topological phenomenon, but a magnetic phenomenon generated by the generation and annihilation of the inhomogeneous magnetic domains. These intriguing results provide new insights into novel physical phenomena in 2D ferromagnets and will play a significant impact on the future development of 2D spintronics.

## 2 Results and Discussion

### 2.1 Magnetic Properties of Bulk and Thin‐Flake Fe_5_GeTe_2_ Crystals

**Figure 1:**
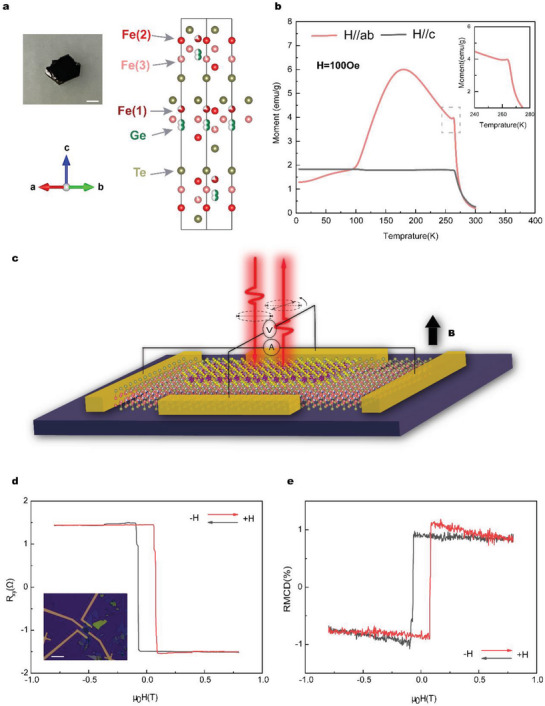
a) Crystal structure of Fe_5_GeTe_2_ and crystal picture. The scale bar is 1 mm. b) Temperature dependence of the magnetization measured under a magnetic field of 100 Oe with the magnetic field parallel (red, *H//ab*) and perpendicular (black, *H//c*) to the sample surface. The inset shows the magnetic anomaly with *H//ab*. c) Schematic diagram of Fe_5_GeTe_2_/MnPS_3_ heterostructure device, RMCD, and magneto‐transport measurement are performed simultaneously to characterize the magnetic properties. d,e) The artificial topology phenomenon of magneto‐transport and RMCD measurement (660 nm) in Fe_5_GeTe_2_/MnPS_3_ heterostructures at 10 K, respectively. The inset shows the image of Fe_5_GeTe_2_/MnPS_3_ heterostructures. The scale bar is 10 µm.

Fe_5_GeTe_2_ belongs to the crystal family of Fe_n_GeTe_2_ (*n* = 3, 4, 5), which has a structure similar to that of Fe_3_GeTe_2._^[^ ^24^ ^]^ **Figure** **1**a shows the atomic structure of the Fe_5_GeTe_2_ crystal, which is composed of Fe–Ge slabs sandwiched by two Te layers. In each unit cell, there are three Fe sites. The Fe_(1)_ site is regarded as a split site and is located either above or below the Ge site, which makes the crystal structure complex and then produces nontrivial spin structures.^[^ ^22^ ^]^ Figure 1b reveals the temperature‐dependent magnetization (*M–T*) of the bulk Fe_5_GeTe_2_ single crystal acquired via a superconducting quantum interference device (SQUID) under an external magnetic field of *H* = 100 Oe. As the temperature decreases, the out‐of‐plane magnetization (*H//c*) exhibits a weak temperature dependence after a rapid increase near *T*_c_. In contrast, the in‐plane magnetization (*H//ab*) under the same conditions exhibits significant enhancement in a certain temperature range. A small spike at ≈*T* = 263 K and a broad cusp at 100–250 K indicate competition among the ferromagnetic exchange interaction, dipolar interaction, magnetic anisotropy, and DMI.^[^ ^22^ ^]^ In addition, the isothermal *M*–*H* curves at different temperatures for both field directions are shown in Figure S1 (Supporting Information). At ≈100 K, the easy magnetization axis switches from the in‐plane (*ab* plane) direction to the out‐of‐plane direction (*c*‐axis). During the whole cooling process, the competition between in‐plane magnetization and out‐of‐plane magnetization is extremely likely to induce spin rearrangement and generate diverse magnetic domain structures.^[^ ^21^ ^]^ While for thin Fe_5_GeTe_2_ flake, the magnetic domain structures seem independent with thermal cycling treating processes, since the RMCD hysteresis curves taken at the same temperatures are almost the same after any thermal cycling treating processes (Figure S2, Supporting Information). Figure 1c shows a schematic diagram of a Fe_5_GeTe_2_/MnPS_3_ heterostructure device, we performed both RMCD and magneto‐transport measurements simultaneously to characterize the magnetic properties. As shown in Figure 1d,e, the hysteresis loops of Fe_5_GeTe_2_/MnPS_3_ (Sample1) measured by transport and RMCD are very close. Anomalous hump‐like features could be observed clearly in both hysteresis curves, which means that the RMCD results could reproduce the transport results perfectly in Fe_5_GeTe_2_ and its heterostructures systems. Therefore, we will use RMCD methods instead of the transport method to investigate the magnetic properties of Fe_5_GeTe_2_ and Fe_5_GeTe_2_/MnPS_3_ in the following context.

### 2.2 Current‐Enhanced and Wavelength‐Dependent Anomalous Hump‐Like Features

**Figure 2:**
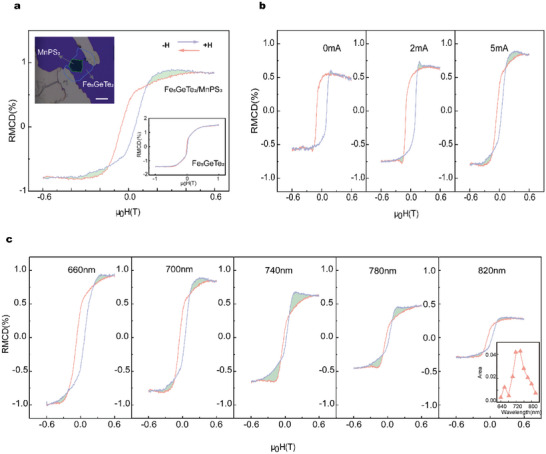
a) Anomalous RMCD in Fe_5_GeTe_2_/MnPS_3_ heterostructures at 10 K with an in‐plane current of 5 mA applied. The green‐shaded area is distinct from the ordinary RMCD signal. The top‐left inset shows a microscope image of the Fe_5_GeTe_2_/MnPS_3_ heterostructure device, and the bottom‐right inset shows the ordinary RMCD of Fe_5_GeTe_2._ The scale bar is 10 µm. b) Current‐dependent anomalous RMCD results at 10 K of 700 nm. c) Anomalous RMCD results as a function of wavelength with an applied in‐plane current of 5 mA at 10 K in Fe_5_GeTe_2_/MnPS_3_. The inset shows the amplitude of the anomalous RMCD with wavelength.

To clearly understand the underlying mechanism of this anomalous hump‐like phenomenon in the Fe_5_GeTe_2_/MnPS_3_ heterostructure, we first performed current‐ and wavelength‐dependent RMCD measurements for a Fe_5_GeTe_2_/MnPS_3_ heterostructure. As shown in **Figure** **2**a, when a 700 nm laser was used to measure the RMCD signal of the Fe_5_GeTe_2_/MnPS_3_ heterostructure with an in‐plane current of 5 mA applied to it, hysteresis loops with hump and dip could be observed (Sample 2), which is quite distinct from the ordinary RMCD signal in Fe_5_GeTe_2_ (the inset of Figure 2a). As the in‐plane current increases from zero to 5 mA, the anomalous hump‐like RMCD signal (marked by the green‐shaded area) becomes obvious (Figure 2b). Moreover, when we fixed the in‐plane current at 5 mA and vary the measurement wavelength, the hump‐like phenomena change accordingly (Figure 2c). The amplitude of the anomalous hump‐like RMCD (defined as the area difference between the peak of the hump and the loop) increases and then decreases with wavelength (inset of Figure 2c). Remarkably, the identical hump‐like phenomena were also observed in the magneto‐optical Kerr effect (MOKE) signal, and the magnitude of its anomalous signal varies with wavelength (More details can be found in Figure S3, Supporting Information). The above results indicate that both current and interface play important roles in this anomalous hump features. Therefore, the effects of antiferromagnetic MnPS_3_ and an in‐plane current on the magnetic properties of Fe_5_GeTe_2_ would be investigated individually in the following context.

### 2.3 Magnetic Modulation of Fe_5_GeTe_2_ and the Fe_5_GeTe_2_/MnPS_3_ Heterostructure

**Figure 3:**
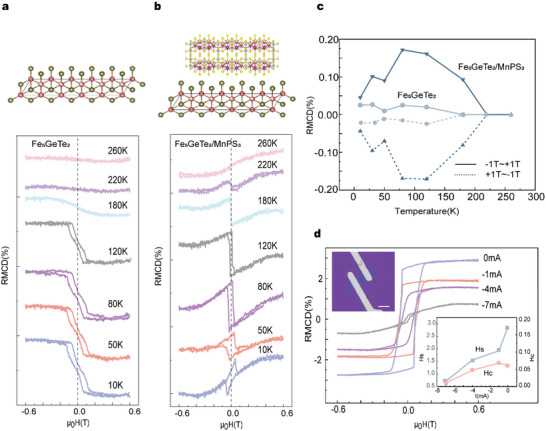
a) Hysteresis loops of a Fe_5_GeTe_2_ flake taken by RMCD in the temperature range of 10–260 K. b) Hysteresis loops of the Fe_5_GeTe_2_/MnPS_3_ heterostructure taken under the same conditions as for Fe_5_GeTe_2_. c) Temperature dependence of the residual magnetism in Fe_5_GeTe_2_ and the Fe_5_GeTe_2_/MnPS_3_ heterostructure. The solid line represents the data taken from the negative field to the positive field, whereas the dashed line represents the data taken from the positive field to the negative field. d) Hysteresis loops of Fe_5_GeTe_2_ taken by RMCD with the in‐plane current varying from 0 to −7 mA. The top‐left inset shows an image of the sample, and the bottom‐right figure shows the current dependence of the *H*_c_ and saturation magnetization (*H*s) at 10 K. The scale bar is 10 µm.

To evaluate the effect of MnPS_3_ on the magnetic properties of Fe_5_GeTe_2_, a three‐step procedure was carried out during the RMCD measurement. First, temperature‐ and wavelength‐dependent RMCD measurements of Fe_5_GeTe_2_ were performed. Then, a piece of MnPS_3_ flake was transferred onto the surface of Fe_5_GeTe_2_. After that, RMCD measurements were performed on the Fe_5_GeTe_2_/MnPS_3_ heterostructure. To check the sample quality of Fe_5_GeTe_2_/MnPS_3_, Raman scattering measurements were taken at the Fe_5_GeTe_2_ and MnPS_3_ flakes and the as‐stacked Fe_5_GeTe_2_/MnPS_3_ heterostructure. The Raman signatures in the stacked area are identical to those of the individual layers, and no additional new peak is observed for the stacked areas (Figure S4c, Supporting Information). During the whole experimental procedure, the samples were placed either in a glove box or in a small sealed copper box filled with nitrogen gas to avoid possible oxidation by air. **Figure** **3**a,b show the temperature‐dependent RMCD results of the Fe_5_GeTe_2_ flake (Sample A ≈ 30 nm) and its Fe_5_GeTe_2_/MnPS_3_ heterostructure (Sample HS‐A), respectively. The incident light is 700 nm with a beam spot of 10 µm and a detection power of 5–10 *µ*W. As indicated in the figure, at temperatures lower than 180 K, a contracted coil‐shaped hysteresis loop is present in Fe_5_GeTe_2._^[^ ^25^, ^26^, ^27^ ^]^ In contrast, the RMCD loop in the Fe_5_GeTe_2_/MnPS_3_ heterostructure is irregular below 260 K, where the RMCD value above the saturation field is much smaller than the maximum value. A more detailed study will be given in the following section. Figure 3c shows the residual magnetism of Fe_5_GeTe_2_ and Fe_5_GeTe_2_/MnPS_3_ as a function of temperature at *μ*_0_ *H* = 0. The residual magnetism of the Fe_5_GeTe_2_/MnPS_3_ heterostructure is several times larger than that of the Fe_5_GeTe_2_ counterpart, which means that the proximity coupling effect of Fe_5_GeTe_2_/MnPS_3_ could significantly enhance the magnetic properties of Fe_5_GeTe_2_.^[^ ^28^ ^]^ Then, a thinner Fe_5_GeTe_2_ flake (Sample B ≈ 20 nm) and its Fe_5_GeTe_2_/MnPS_3_ heterostructure (Sample HS‐B) were fabricated and comparatively investigated. The results are consistent with the previous results, where the coercive field (*H*_c_) of Fe_5_GeTe_2_ is significantly enhanced by the interface of Fe_5_GeTe_2_/MnPS_3_ heterostructures (Figure S5, Supporting Information). Moreover, when MnPS_3_ was removed, the coercive field reverted to the original value (Figure S6, Supporting Information). In addition to interface engineering,^[^ ^29^ ^]^ an in‐plane current application could also modulate the magnetism of metallic ferromagnets.^[^ ^30^, ^31^ ^]^ As the current changes from 0 to −7 mA, the hysteresis loop of Fe_5_GeTe_2_ shrinks and nearly disappears at ≈−7 mA. As clarified in the bottom‐right inset of Figure 3d, when the in‐plane current is applied to Fe_5_GeTe_2_, *H*_c_ and the saturation field (*H*_s_) gradually decrease as the current increases. This indicates that the in‐plane current could drive the motion of the domain walls and disturb the magnetic order of Fe_5_GeTe_2_, effectively weaken its magnetic anisotropy.

The above studies illustrate that interface engineering and in‐plane current application are two effective ways to modulate the magnetic anisotropy of Fe_5_GeTe_2_ in opposite directions, the influences of the interface effect and the current on magnetic domains are not negligible. Thus, it is very possible to generate chiral spin structures and a hump‐like feature. However, it may not be appropriate to equate it with the topological Hall signal simply by virtue of this hump feature, which may also be generated by the superposition of multiple magnetic signals. Therefore, we need more experimental data to confirm our speculations on this controversial issue in the field.

### 2.4 The Artificial Topological Phenomena in Fe_5_GeTe_2_/MnPS_3_ Heterostructure

**Figure 4:**
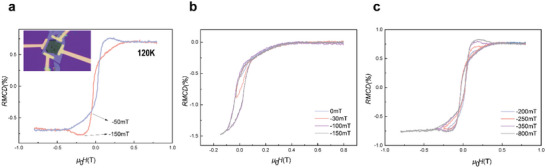
a) The anomalous RMCD in Fe_5_GeTe_2_/MnPS_3_ heterostructures at 120 K with 0.5 mA current applied to it. b) The minor loops within the field of −150 mT, stopping at 0, −30, −100, and −150 mT. c) The minor loops beyond the field of −150 mT, stopping at −200, −250, −350, and −800 mT.

To distinguish whether the hump‐like feature is a genuine topological phenomenon, the measurement of minor loops was performed.^[^ ^32^ ^]^ For the genuine THE, the appearance of dips and humps in the minor loops does not depend on the history of the sweeping magnetic field, while the opposite occurs for the artificial ones. **Figure** **4**a shows the anomalous hump‐like RMCD in the Fe_5_GeTe_2_/MnPS_3_ heterostructure (Sample 3) at 120 K with 0.5 mA current applied to it. The minor loops were taken by sweeping the magnetic field from +800 mT to zero or a certain value of negative field (0, −30, −100, −150, −200, −250, −300, −800 mT) and then back to +800 mT. As presented in Figure 4b, minor loops within the field of ‐150 mT, do not reach the field range of the dips at the negative field and show no humps at positive fields. When the magnitude of the negative magnetic field is beyond the value of −150 mT, the dips at the negative field and the humps at positive fields develop gradually simultaneously (Figure 4c), and the emergence of the dips or humps depends on how the minor loops are scanned. It means that the hump‐like feature cannot be in line with the story of the skyrmions formations. Therefore, the hump‐like feature here may be attributed to the current‐induced inhomogeneous generation of magnetic domains at the interface and the Fe_5_GeTe_2_ body part, which have the opposite RMCD signals. In addition, for the irregular hysteresis loop shown in Figure 3b, although this irregular loop is slightly different from the artificial topological phenomenon mentioned above, it may arise from a similar mechanism. Proceeding from this, we have a reasonable explanation for this irregular loop to the hump‐like evolution process

**Figure 5:**
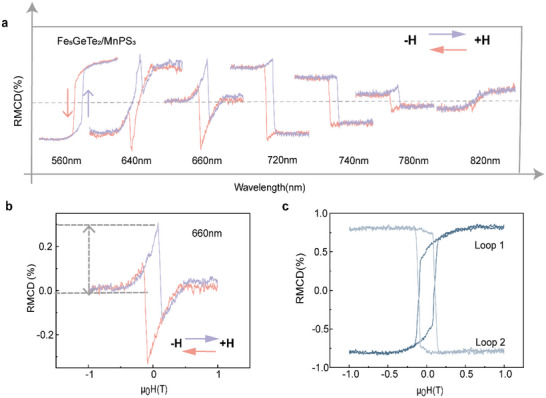
a) Wavelength‐dependent RMCD signal from 560 nm to 840 nm at 10 K. b,c) Typical irregular RMCD signal at 660 nm and corresponding decomposed components.

To clarify the origin of the irregular RMCD signal, wavelength‐dependent RMCD measurements were taken at wavelengths ranging from 500–900 nm at 10 K for Sample HS‐B (thickness of Fe_5_GeTe_2_ is ≈20 nm). **Figure** **5**a shows the hysteresis loops of the Fe_5_GeTe_2_/MnPS_3_ heterostructure (Sample HS‐B) taken at various wavelengths under 10 K. For the hysteresis curve taken at 560 nm, the loop shape is regular, whereas for the data taken at 640–820 nm, the loop shapes are irregular. As the wavelength changes, the shape varies accordingly. To rule out the possible effect of electron transfer between MnPS_3_ and Fe_5_GeTe_2_, the absorption spectrum of MnPS_3_ (550–600 nm) and excitation power‐dependent RMCD of the heterostructures were characterized (Figure S7, Supporting Information), and no obvious relationship between the absorption and RMCD was observed. Moreover, an alternative explanation for humps is that the non‐trivial coupling of circularly polarized light to MnPS_3_ is also ruled out. The spectrally resolved measurement of RMCD for MnPS_3_ was provided in Figure S8 (Supporting Information), no humps emerged. As a comparison, Fe_3_GeTe_2_/MnPS_3_ heterostructures were also prepared and measured in the same way as Fe_5_GeTe_2_/MnPS_3_ and no irregular hysteresis loop was observed (the results are presented in Figure S9, Supporting Information). Considering that irregular hysteresis loops only appear in Fe_5_GeTe_2_/MnPS_3_ heterostructures (the results of Fe_5_GeTe_2_/MnPS_3_ heterostructures (sample HS‐A), Fe_5_GeTe_2_ (Sample B ≈ 20 nm) and Fe_5_GeTe_2_ (Sample A ≈ 30 nm) are given in the Figures S10–S12, Supporting Information), it is reasonable to conclude that the interface and complex magnetic domain structure of Fe_5_GeTe_2_ play an important role in generating such irregular hysteresis loops.^[^ ^33^, ^34^, ^35^ ^]^ And as shown in Figure 5b,c, the irregular hysteresis loops taken at 660 nm were successfully split into two regular loops with opposite signs, originating from the interface and Fe_5_GeTe_2_. Due to Kramers–Kronig relations,^[^ ^36^ ^]^ the RMCD intensity in these two regions is wavelength dependent and responds differently, thus leading to a change in the hysteresis shape. Then, when in‐plane current acts on the Fe_5_GeTe_2_/MnPS_3_ heterostructures, the influence of the current on the magnetic domains in the two regions is not uniform, so the RMCD signal in Figure 5b may gradually develop into Figure 4a.

## 3 Conclusion

In summary, the artificial topology phenomenon in Fe_5_GeTe_2_/MnPS_3_ heterostructure has been investigated by using the RMCD technique. The formation of inhomogeneous magnetic domains at the interface and in the Fe_5_GeTe_2_ is responsible for the hump‐like feature rather than THE. Furthermore, we point out that interfacial effects and the current manipulation could tune the magnetic properties of 2D vdW materials. Our work provides a deeper understanding of topological phenomena and may promote the development of skyrmion‐based devices in 2D magnetic materials.

## 4 Experimental Section

### Growth of Single‐Crystals

The MnPS_3_ single crystal was grown by chemical vapor transport method, powders of Mn (99.99%), P (99.999%), and S (99.999%) in a 1:1:3 ratio were used and ground uniformly in an N_2_‐protection glove box. The mixture was then sealed into a quartz ampule with appropriate iodine. Finally, the ampule was placed into a horizontal two‐zone furnace with a hot zone at 953 K and a cold zone at 873 K for eight days and cooled with 3 K min^−1^ to room temperature. The Fe_5_GeTe_2_ Single‐crystal was purchased from Shanghai Onway Technology Co., Ltd. The crystal structure of both crystals was checked by X‐ray diffraction (XRD).

### Fabrication of Fe_5_GeTe_2_/MnPS_3_ Heterostructures

Mechanical exfoliation methods were used to prepare Fe_5_GeTe_2_ and MnPS_3_ thin flakes, dry transfer method was used to fabricate Fe_5_GeTe_2_/MnPS_3_ heterostructures. To prepare the Fe_5_GeTe_2_/MnPS_3_ heterostructure, Fe_5_GeTe_2_ and MnPS_3_ crystals on polydimethylsiloxane and selected flakes with suitable thickness were first mechanically exfoliated under a microscope. Then, the multilayer Fe_5_GeTe_2_ flakes were transferred onto Si/SiO_2_ substrates, and an MnPS_3_ flake was stacked on Fe_5_GeTe_2_ by the dry transfer method. The whole process was performed in a glovebox, and the sample was sealed in a copper box during measurement, thus avoiding oxidation due to exposure to air.

### Characterization of the Single Crystals and the Heterostructures

The XRD and SQUID were used to characterize the quality, lattice orientation, and magnetic properties of Fe_5_GeTe_2_ and MnPS_3_ single crystals (Figure S13, Supporting Information). Raman measurements were performed to monitor the degradation and strain‐induced lattice disturbance of Fe_5_GeTe_2_ and MnPS_3_ thin flakes. The cross–sectional high–angle annular dark–field–based scanning transmission electron microscopy (HAADF–STEM) was performed to check the interfacial structures of Fe_5_GeTe_2_/MnPS_3_ heterostructures (Figure S14, Supporting Information).

### Electrode Fabrication

After the heterostructure was prepared, the laser direct writing method (MicroWriter Baby, Durham Magneto Optics Ltd.) was used to fabricate Cr/Au (10/90 nm) electrodes through electron beam evaporation. After the lift‐off process, electrodes were obtained. In the preparation of completely oxidation‐free samples, Cr/Au electrodes were first prepared on a Si/SiO_2_ substrate, and Fe_5_GeTe_2_ and MnPS_3_ thin flakes were sequentially transferred onto the prepatterned electrodes via a dry transfer method in a glovebox. Then, exfoliated h‐BN was stacked on the Fe_5_GeTe_2_/MnPS_3_ device to prevent oxidation degradation.

### Reflective Magnetic Circular Dichroism (RMCD) and Magneto–Optical Kerr Effect (MOKE) Measurements

RMCD and MOKE measurements were performed in a superconductive magnet (Oxford Instruments) with a temperature range of 300–4.2 K and an out‐of‐plane magnetic field up to 5 T. The light source had a wavelength range from 500–900 nm. The signal was collected by a pre‐amplified photodetector connected to three lock‐in amplifiers (LIAs).^[^ ^28^ ^]^

### Electrical Measurement

The electrical measurement module was integrated with the RMCD measurement setup, and RMCD, MOKE, and magneto‐transport measurements could be conducted simultaneously under the same experimental conditions. Detailed information about the setup is given in Figure S15 (Supporting Information). During the electrical measurements, an AC current with the frequency of 13–100 Hz and an amplitude of 1–200 µA was supplied by a Keithley 6221 AC source, and the Hall response was monitored by a Stanford SR830 lock‐in amplifier.

## Conflict of Interest

The authors declare no conflict of interest.

## Supporting information

Supporting Information

## Data Availability

The data that support the findings of this study are available from the corresponding author upon reasonable request.
