# Supplementary material for: Manipulation and Optical Detection of Artificial Topological Phenomena in 2D Van der Waals Fe5GeTe2/MnPS3 Heterostructures
Source: Adv Sci (Weinh). 2023 Jun 16;10(22):2207617. doi: 10.1002/advs.202207617 (PMC10401167; doi:10.1002/advs.202207617)
Supplement: Supplementary file 1 — Supporting Information [file ADVS-10-2207617-s001.pdf]

ADVANCED SCIENCE
Open Access

Supporting Information

for *Adv. Sci.*, DOI 10.1002/advs.202207617

Manipulation and Optical Detection of Artificial Topological Phenomena in 2D Van der Waals $Fe_5GeTe_2/MnPS_3$ Heterostructures

Xiaodie Chen, Haoyun Wang, Manshi Li, Qinghua Hao, Menghao Cai, Hongwei Dai, Hongjing Chen, Yuntong Xing, Jie Liu, Xia Wang, Tianyou Zhai, Xing Zhou and Jun-Bo Han**

Supporting Information

Manipulation and Optical Detection of Artificial Topological Phenomena in Two-Dimensional van der Waals $Fe_5GeTe_2/MnPS_3$ Heterostructures

Xiaodie Chen, Haoyun Wang, Manshi Li, Qinghua Hao, Menghao Cai, Hongwei Dai, Hongjing Chen, Yuntong Xing, Jie Liu, Xia Wang, Tianyou Zhai, Xing Zhou, Jun-Bo Han**

Dr. X. Chen, Mr. M. Li, Dr. Q. Hao, Dr. M. Cai, Dr. H. Dai, Dr. H. Chen, Dr. Y. Xing, Prof. J. B. Han
Wuhan National High Magnetic Field Center and Department of Physics,
Huazhong University of Science and Technology, Wuhan, 430074, P. R. China
E-mail: *junbo.han@mail.hust.edu.cn* (Jun-Bo Han)

Dr. H. Wang, Dr. J. Liu, Prof. T. Zhai, Prof. X. Zhou
State Key Laboratory of Materials Processing and Die & Mould Technology, School of Materials Science and Engineering, Huazhong University of Science and Technology, Wuhan, 430074, P. R. China
E-mail: *zhoux0903@hust.edu.cn* (Xing Zhou)

Prof. X. Wang
School of Elementary Education, Wuhan City Polytechnic College, Wuhan, 430074, P. R. China

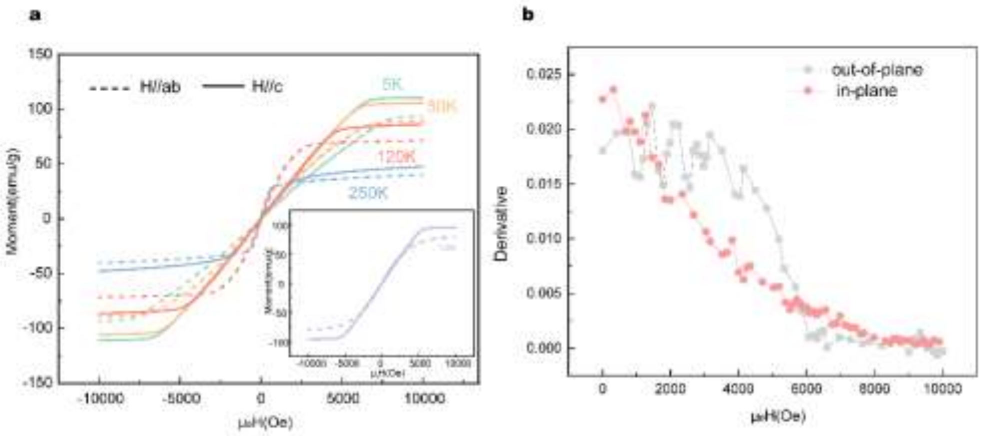

Figure S1. (a) Magnetic field dependence of magnetization *M* ($\mu_0 H$) for *H//c* and *H//ab* at various temperatures. The inset of (a) shows the *M-H* curves at 100 K for both field directions. (b) The first-order derivative results of *M-H* curves at 100 K for both field directions. The results indicate that the out-of-plane magnetization reaches saturation more quickly, suggesting that the easy magnetization axis is the c-axis.

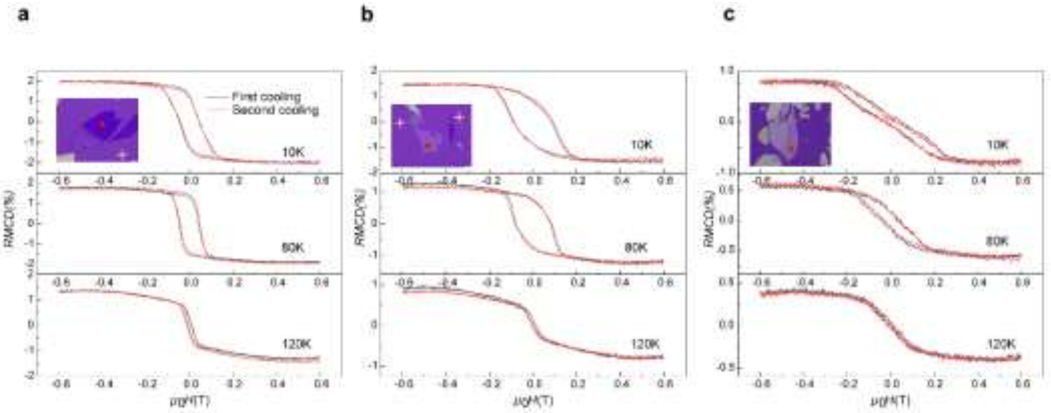

Figure S2. Temperature-dependent RMCD results of Fe_5GeTe_2 thin flakes with different thicknesses at 700nm.

To study the effect of thermal-cycling process on the magnetic signals of thin-layer Fe_5GeTe_2 flakes, three Fe_5GeTe_2 flakes with different thicknesses were

prepared and measured at different thermal-cycling treating processes. The RMCD hysteresis loops of each sample coincide completely for any given temperatures (See Figure S2). The results demonstrate that for thin-layer of Fe_5GeTe_2, thermal cycling does not alter the magnetic order.

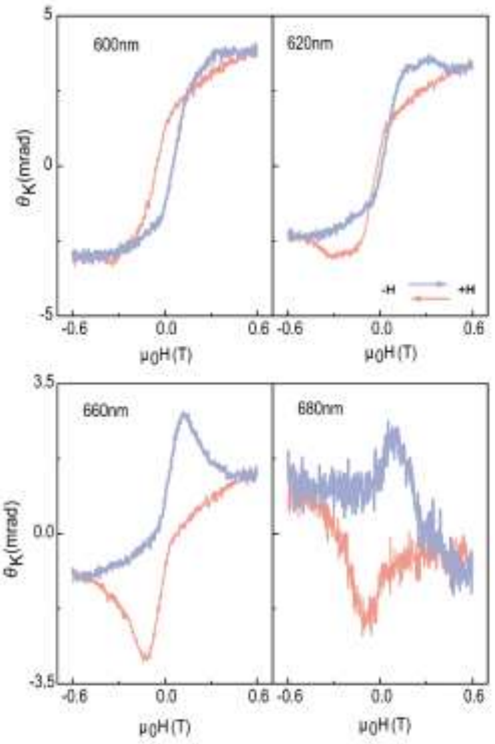

Figure S3. Wavelength-dependent MOKE results of $Fe_5GeTe_2/MnPS_3$ heterostructure (Sample 2) taken at 10K with a 5 mA in-plane current applied on it.

Figure S3 shows the wavelength-dependent MOKE results of $Fe_5GeTe_2/MnPS_3$ heterostructure (Sample 2), which is corresponding to the RMCD data in Figure 2c. As the MOKE signal (θ_K) and RMCD signal (ε_K) are a pair of optical parameters that are connected by the real part and imaginary part of the complex polar Kerr rotation function, the identical hump-like phenomena shown in RMCD could also

observed in MOKE curves.

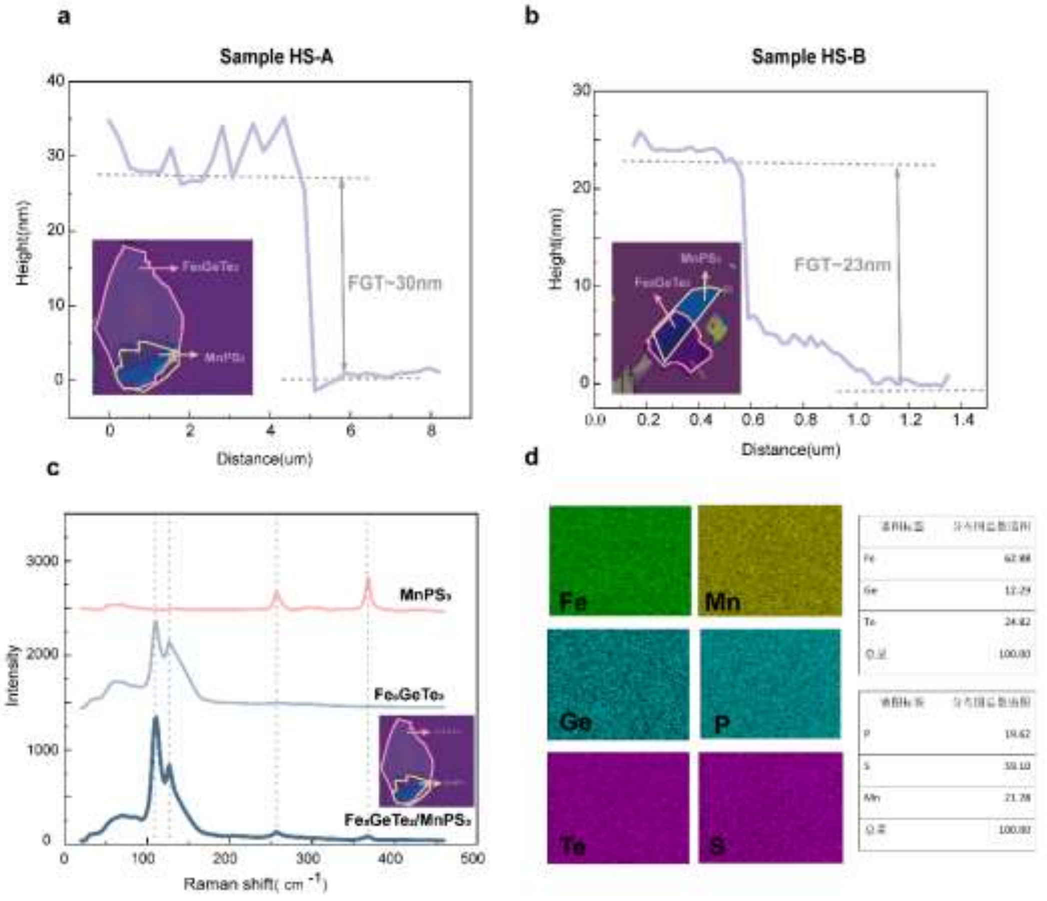

Figure S4. Basic characterization of $Fe_5GeTe_2/MnPS_3$ heterostructures (sample HS-A and sample HS-B). (a) Optical image and height curve of sample HS-A; b) Optical image and height curve of sample HS-B; (c) Raman spectra of $MnPS_3$ (pink), Fe_5GeTe_2 (blue), and $Fe_5GeTe_2/MnPS_3$ heterostructure (dark Blue). The inset shows the microscope image of $Fe_5GeTe_2/MnPS_3$ heterostructure. The Raman signatures in the stacked area are identical to those of the individual layers, and no additional new peaks was observed for the stacked areas; it means that the possible strain during the transfer processes does not have a remarkable effect on the lattice structure of the $Fe_5GeTe_2/MnPS_3$ heterostructure. (d) EDS analysis of Fe_5GeTe_2 and $MnPS_3$ single

crystals.

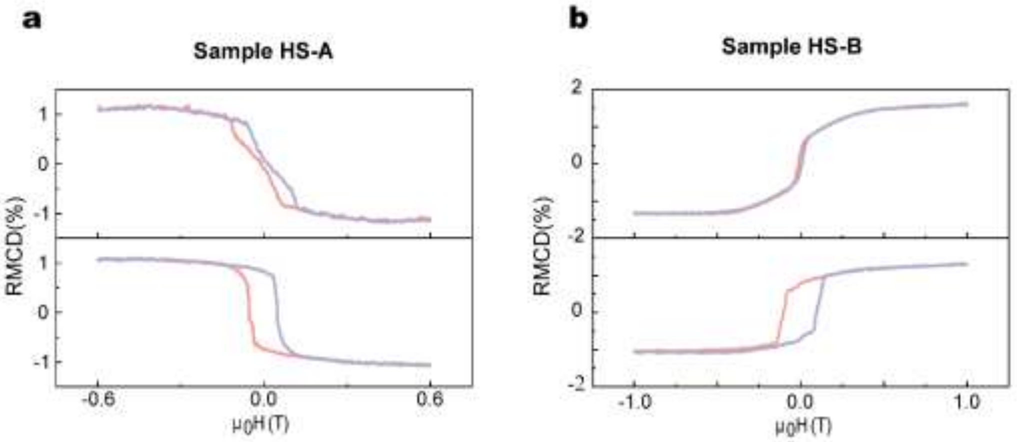

Figure S5. Comparison of coercive fields between $Fe_5GeTe_2/MnPS_3$ (bottom panels) and pure Fe_5GeTe_2 (top panels)。(a) Hysteresis loops of sample HS-A taken at 10 K with the measurement wavelength of 560 nm, where the thickness of Fe_5GeTe_2 is 30 nm; (b) Hysteresis loops of sample HS-B at the same condition as HS-A, the thickness of Fe_5GeTe_2 in HS-B is 20 nm.

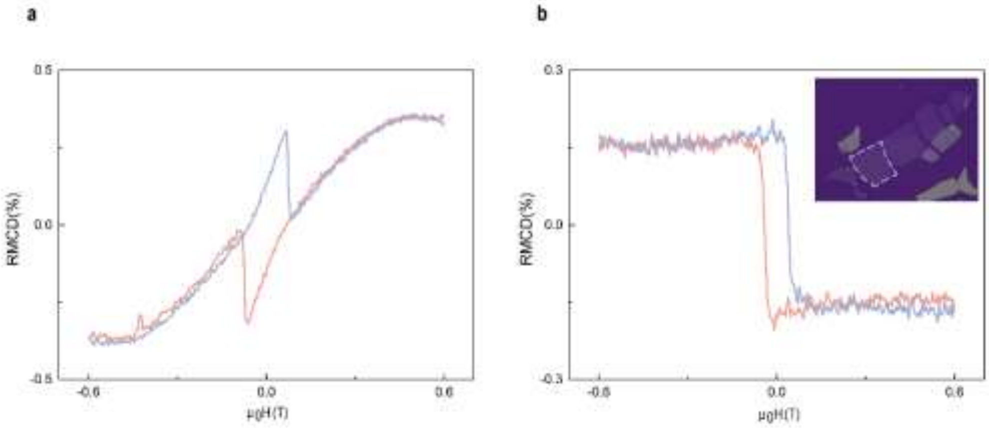

Figure S6. RMCD of $Fe_5GeTe_2/MnPS_3$ before and after $MnPS_3$ was removed. (a) RMCD result of $Fe_5GeTe_2/MnPS_3$; (b) RMCD Fe_5GeTe_2 after $MnPS_3$ was removed from its heterostructure. The RMCD measurements were taken at 10 K with the

wavelength of 720 nm.

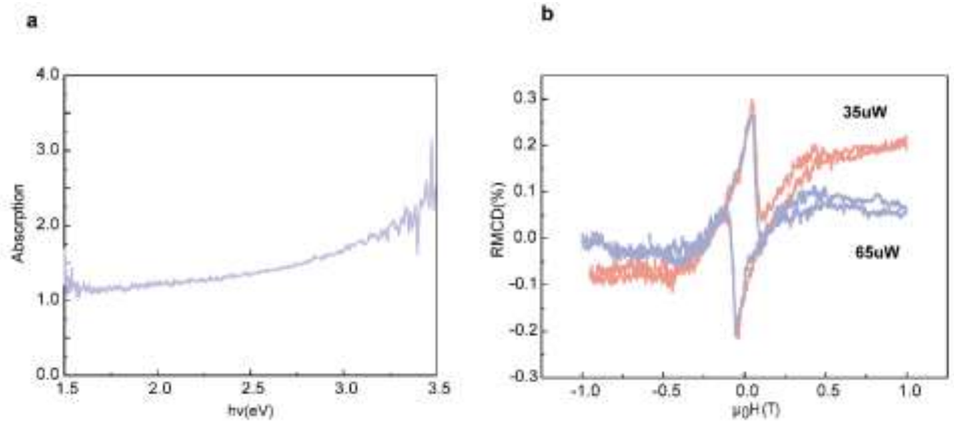

Figure S7. (a) Absorption spectrum of $MnPS_3$; (b) Excitation power dependent RMCD of the heterostructures taken with the excitation power of 35 μW and 65 μW, respectively. There is no distinct difference in the magnitude of the RMCD signal at different excitation powers, indicating that the electron transfer between $MnPS_3$ and Fe_5GeTe_2 is not responsible for the hump-like phenomenon.

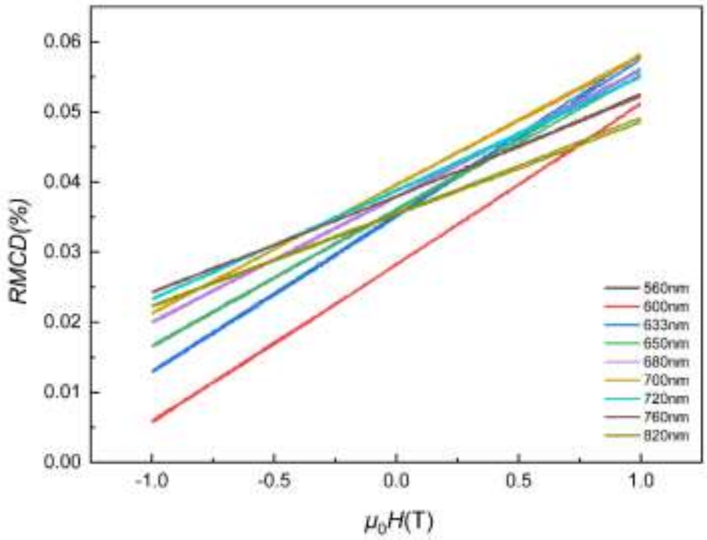

Figure S8. Spectrally resolved measurement of RMCD for $MnPS_3$. No hump or dip has been observed in the magnetic dependent RMCD of $MnPS_3$ at different wavelengths, which excludes the non-trivial coupling of circularly polarized light to

$MnPS_3$.

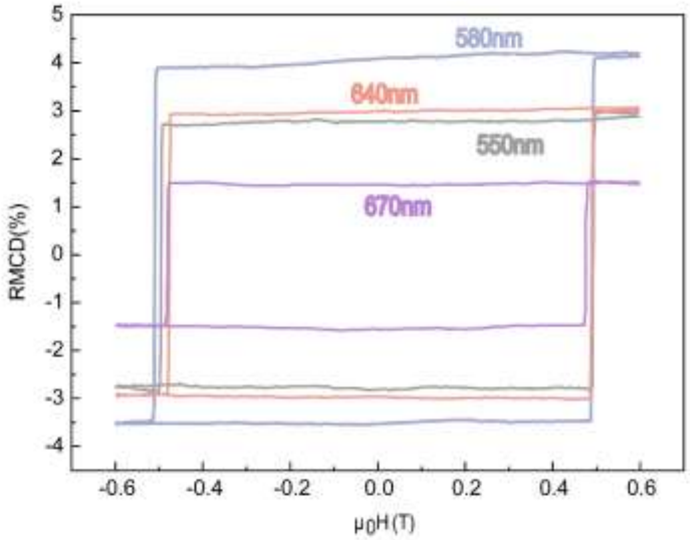

Figure S9. Wavelength-dependent RMCD results of Fe_3GeTe_2/$MnPS_3$ heterostructure.

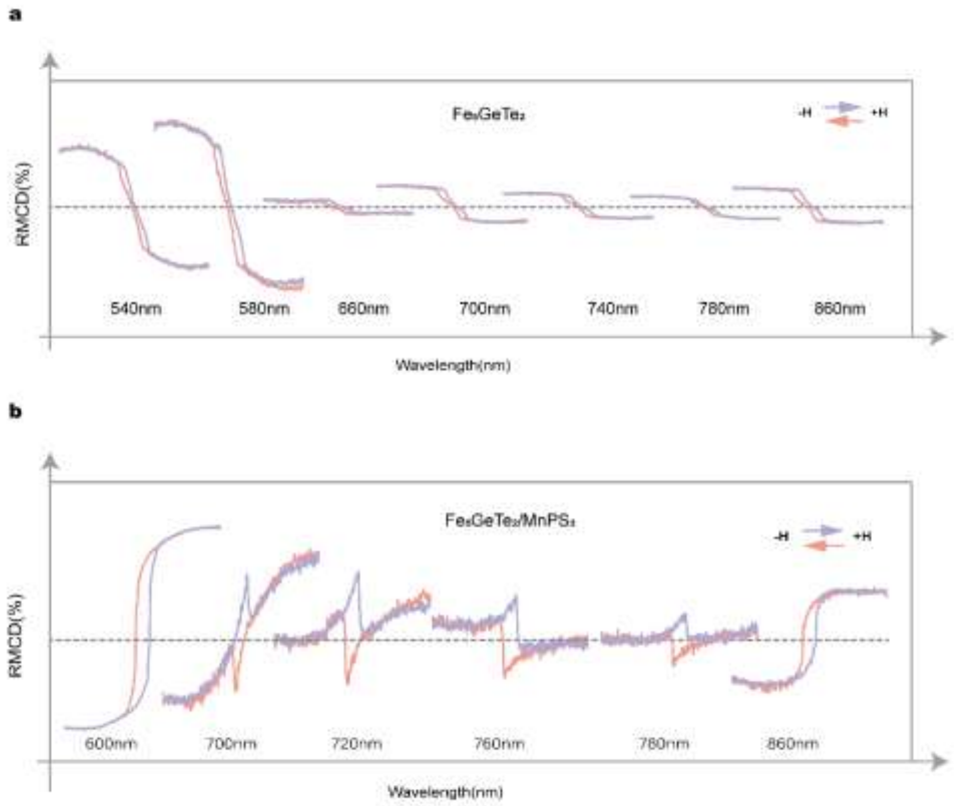

Figure S10. Wavelength-dependent RMCD results of (a) Fe_5GeTe_2 (Sample A~30 nm) and (b) $Fe_5GeTe_2/MnPS_3$ heterostructure (Sample HS-A) at 10 K.

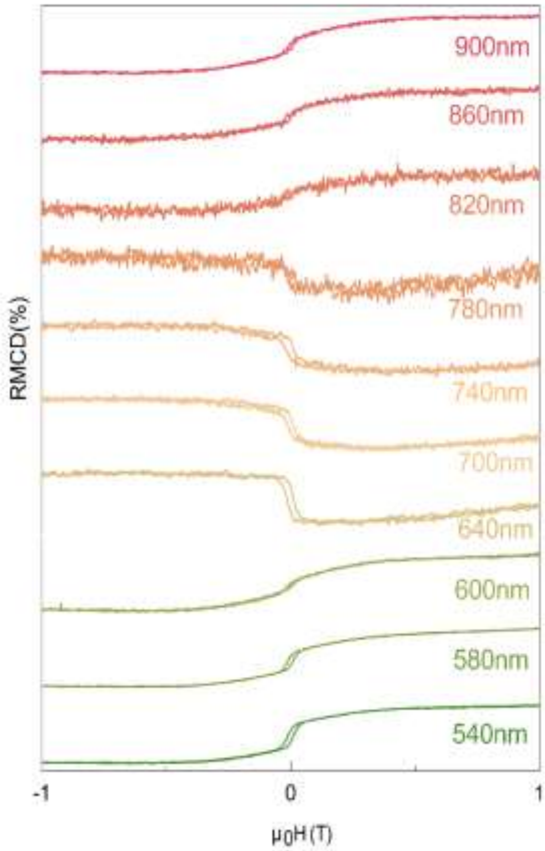

Figure S11. Wavelength-dependent RMCD results of Fe_5GeTe_2 (Sample B) measured at 10 K, the thickness is ~20 nm.

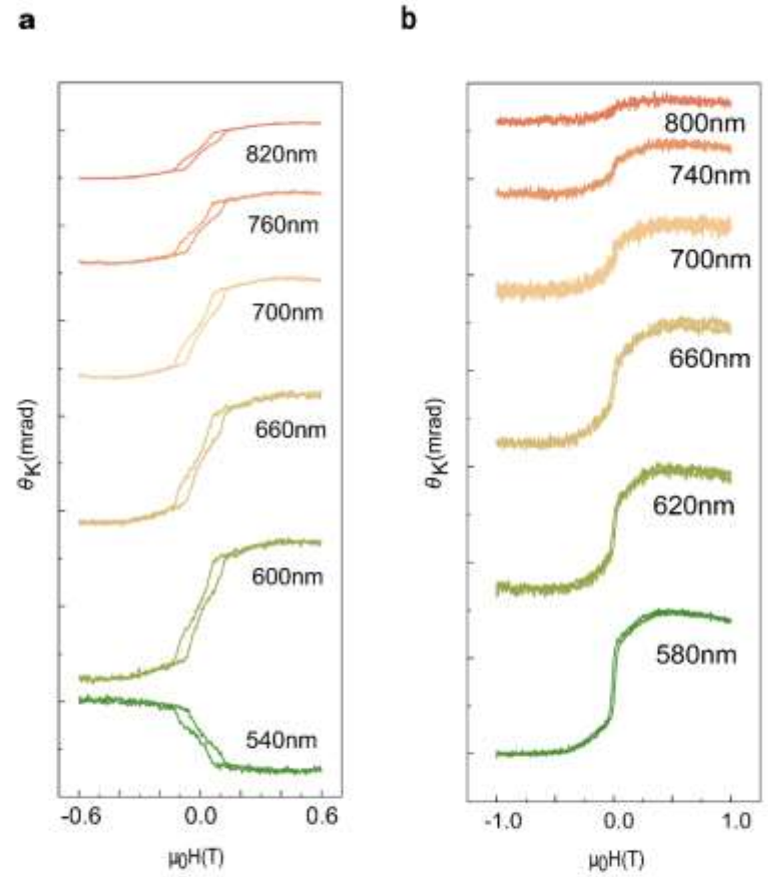

Figure S12. Wavelength-dependent MOKE results of two different Fe_5GeTe_2 flakes measured under the temperature of 10 K. (a) Fe_5GeTe_2 flake with the thickness of 30 nm (Sample A). (b) Fe_5GeTe_2 flake with the thickness of 20 nm (Sample B).

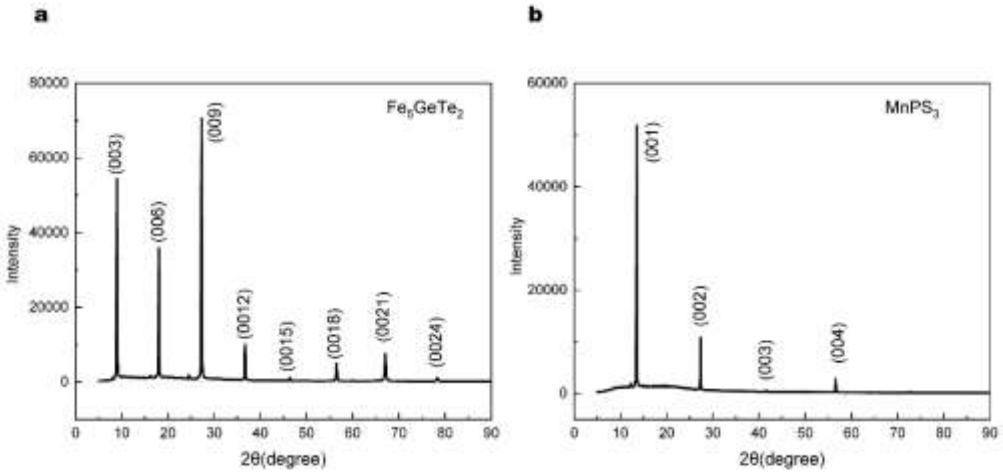

Figure S13. X-ray diffraction (XRD) spectra of Fe_5GeTe_2 (a) and $MnPS_3$ (b) single crystals. The diffraction peaks of both crystals are very sharp which mean that both

crystals are in high quality. The XRD spectra of the Fe_5GeTe_2 single crystal indicates that the c axis is normal to the slab surface.

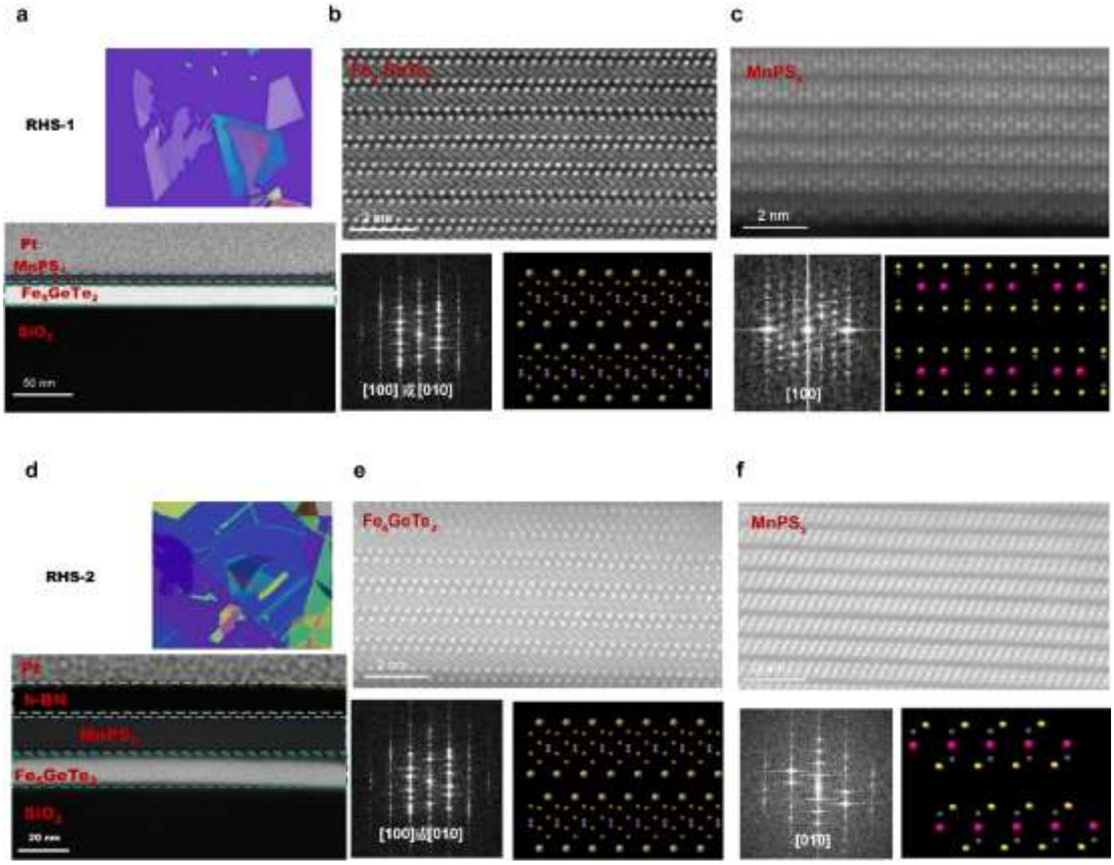

Figure S14. Cross-sectional HAADF STEM images of two Fe_5GeTe_2/$MnPS_3$ heterostructures (RHS-1 and RHS-2). (a) Optical and cross-sectional HAADF STEM images of sample RHS-1. (b) Cross-sectional diffraction pattern and atomic structure of $MnPS_3$ along the [100] direction. (c) Cross-sectional diffraction pattern and atomic structure of Fe_5GeTe_2 along the [100] or [010] directions. (d) Optical and cross-sectional HAADF STEM images of sample RHS-2. (e) Cross-sectional diffraction pattern and atomic structure of $MnPS_3$ along the [010] direction. (f) Cross-sectional diffraction pattern and atomic structure of Fe_5GeTe_2 along the [100] or [010] directions.

Figure S14 show cross-sectional HAADF scanning transmission electron microscopy (STEM) images of two Fe_5GeTe_2/$MnPS_3$ heterostructures (RHS-1 and RHS-2). A clean interface can be observed between the Fe_5GeTe_2 and $MnPS_3$ layers, and different kinds of stacking ways, $MnPS_3$ [100] on Fe_5GeTe_2 ([100] or [010]) (Figure S14a-c) and $MnPS_3$ [010] on Fe_5GeTe_2 ([100] or [010]) (Figure S14d-f), have been observed. Though the stacking ways are different, no obvious difference has been observed between the two different heterostructures.

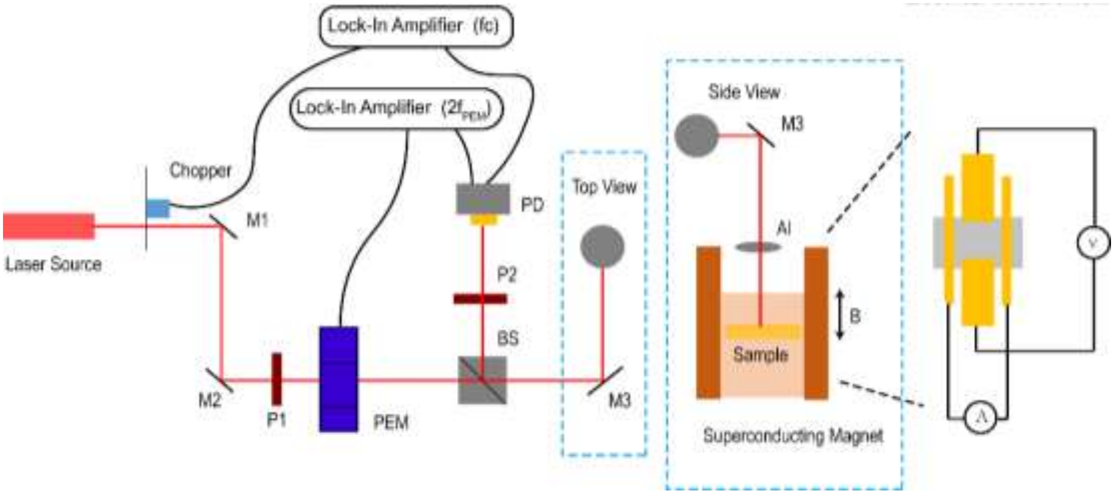

Figure S15. Schematic of the optical setup for Polar Magneto-optical Kerr effect and electrical transport measurements. M1-M3 are mirrors, BS is beam splitter: P1-P2 are linear Polarizers, Al is aspheric lens, PD is photodiode. Electrical Measurement: AC current with the frequency of 13-100 Hz and the amplitude of 1-200 uA was supplied by a Keithley 6221 AC power source. The Hall response was monitored by using a Stanford SR830 lock-in amplifier.
